# Transition-Metal Composition, Optical Absorption, and Channel-Water Characteristics of Natural V-Rich Beryl from the North Muzart River Area, Xinjiang, China

**DOI:** 10.3390/ma19173625

**Published:** 2026-08-26

**Authors:** Tianqi Zhu, Geng Li, Fabian Schmitz

**Affiliations:** 1 School of Gemology, China University of Geosciences (Beijing), Beijing 100083, China; 2109240024@email.cugb.edu.cn; 2 Rhein Main Gem Consulting, Forsterstraße 4, 55118 Mainz, Germany; fabian.d.schmitz@googlemail.com

**Keywords:** natural V-rich beryl, transition-metal composition, optical absorption, channel water, vibrational spectroscopy

## Abstract

Natural V-rich green beryl from the North Muzart River area, Xinjiang, was investigated using microscopy, EDXRF, UV–Vis–NIR, FTIR, and Raman spectroscopy. Ten specimens were examined microscopically; selected detached crystals were analyzed spectroscopically. Thirteen EDXRF analyses on seven detached crystals yielded V_2_O_5_- and Fe_2_O_3_-equivalent contents of 0.562–4.616 wt.% and 0.172–0.494 wt.%, respectively; Cr_2_O_3_ was not detected under the present analytical conditions, and normalized Fe_2_O_3_–V_2_O_5_–Cr_2_O_3_ compositions plot near the V_2_O_5_ endmember. Broad absorption bands near 429 and 617 nm fall within the spectral range commonly attributed to V^3+^ in beryl. Given the V-dominant EDXRF composition, these bands are interpreted as predominantly V-related; however, the V^3+^ assignment remains indirect because EDXRF does not determine the oxidation state, crystallographic site, or local coordination of V, and a minor contribution from Cr below the EDXRF detection capability cannot be excluded. Weaker responses near 366, 391, and 847 nm may involve Fe-related centers. Raman spectra from two selected green regions are consistent with the beryl host, and FTIR spectra of four selected crystals indicate predominantly type-II channel-water features with weaker type-I contributions. These samples provide a natural comparative system for evaluating V-related absorption in beryl and for comparison with V-doped synthetic beryl.

## 1. Introduction

Beryl is a hexagonal cyclosilicate with the ideal formula of Be_3_Al_2_Si_6_O_18_. Its structure consists of six-membered [SiO_4_] rings stacked along the c-axis and linked by AlO_6_ octahedra and BeO_4_ tetrahedra, forming continuous structural channels parallel to the c-axis. These channels can accommodate H_2_O; CO_2_; and non-framework constituents such as Na, Li, K, Rb, and Cs [1,2,3,4,5,6,7]. Transition metals including Cr, V, and Fe can enter or modify local coordination environments in the beryl lattice and produce characteristic absorption in the visible and near-infrared regions [8,9,10,11,12]. UV–Vis–NIR spectroscopy probes transition-metal-related electronic absorption, whereas Raman and FTIR spectroscopy provide complementary information on framework vibrations and channel constituents [13,14,15,16,17].

The green color of emerald is commonly associated with Cr^3+^ and V^3+^. In near-octahedral coordination, electronic transitions of both ions produce broad bands in the violet–blue and orange–red regions, with substantial overlap between their principal absorption ranges [8,18,19,20,21]. Fe^3+^ and Fe^2+^ may add responses in the near-ultraviolet, short-wavelength visible, and near-infrared regions, further complicating spectral assignments in natural samples [9,10,11,12,22,23]. Band positions alone are therefore generally insufficient to distinguish the relative contributions of V and Cr, and independent compositional constraints are required. Artificially doped crystals allow specific dopant-related centers to be examined under controlled conditions, whereas natural crystals commonly contain compositional heterogeneity, fractures, inclusions, and several coexisting trace constituents [24,25,26,27].

In addition to transition-metal composition, the beryl framework and molecular species within its structural channels form an important part of the spectroscopic background. Raman spectroscopy characterizes framework vibrations involving Al–O, Si–O–Si, and Si–O/Be–O units and can verify the dominant phase in the analyzed region. FTIR spectroscopy identifies both framework vibrations and bands associated with type-I and type-II channel water. Natural and synthetic beryl may differ in their combinations of transition-metal constituents, alkali elements, and channel water [5,7,15,16,17,28,29].

Natural green beryl has recently been identified in the North Muzart River area in Zhaosu County, Xinjiang, northwestern China, but this material has not previously been examined through an integrated comparison of transition-metal composition, electronic absorption, framework vibrations, and channel-water features. The samples are enriched in V, whereas Cr was not detected under the present EDXRF conditions, providing a V-dominant compositional context for examining visible absorption.

This study combines microscopy, EDXRF, UV–Vis–NIR, Raman, and FTIR spectroscopy to characterize the transition-metal composition, electronic absorption, dominant phase, and channel-water features of natural V-rich green beryl from the North Muzart River area. Particular emphasis is placed on the compositional constraints on the broad absorption bands near 429 and 617 nm and on the beryl framework and channel environment in which these electronic absorptions occur.

## 2. Materials and Methods

**Figure 1:**
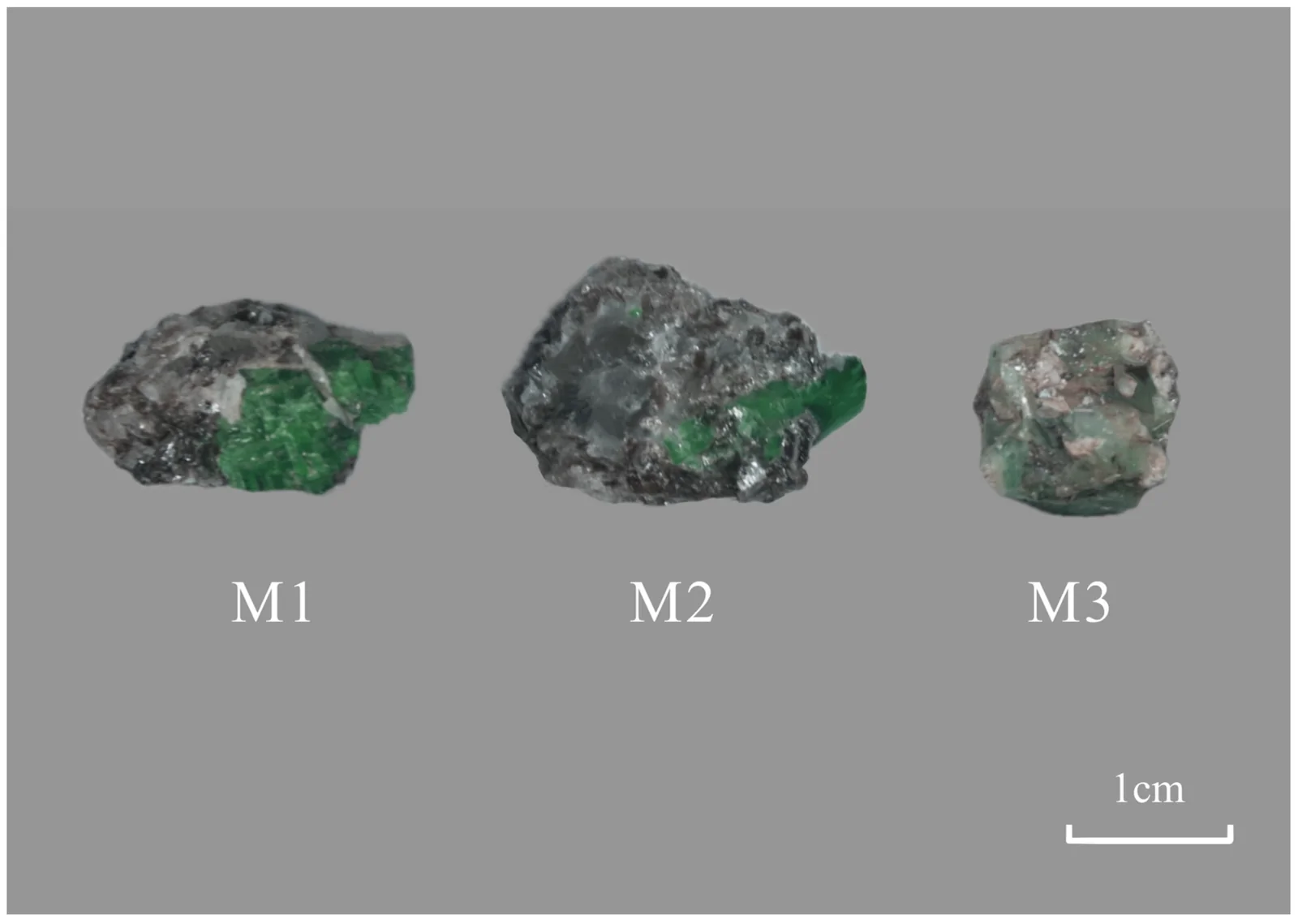
Natural V-rich green beryl specimens attached to host rock from the North Muzart River area (M1–M3).

**Figure 2:**
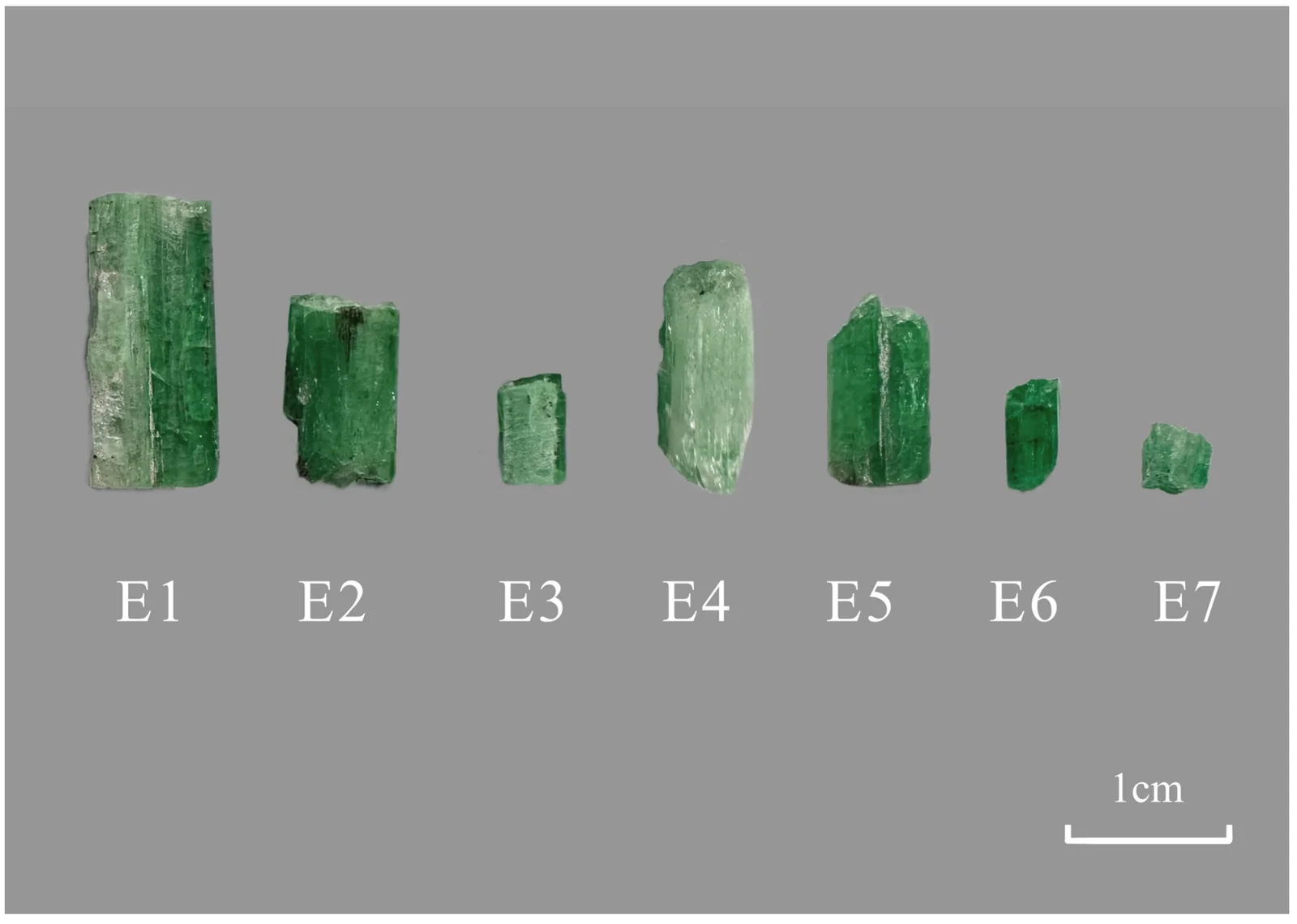
Detached natural V-rich green beryl crystals used for compositional and spectroscopic analyses (E1–E7).

**Figure 3:**
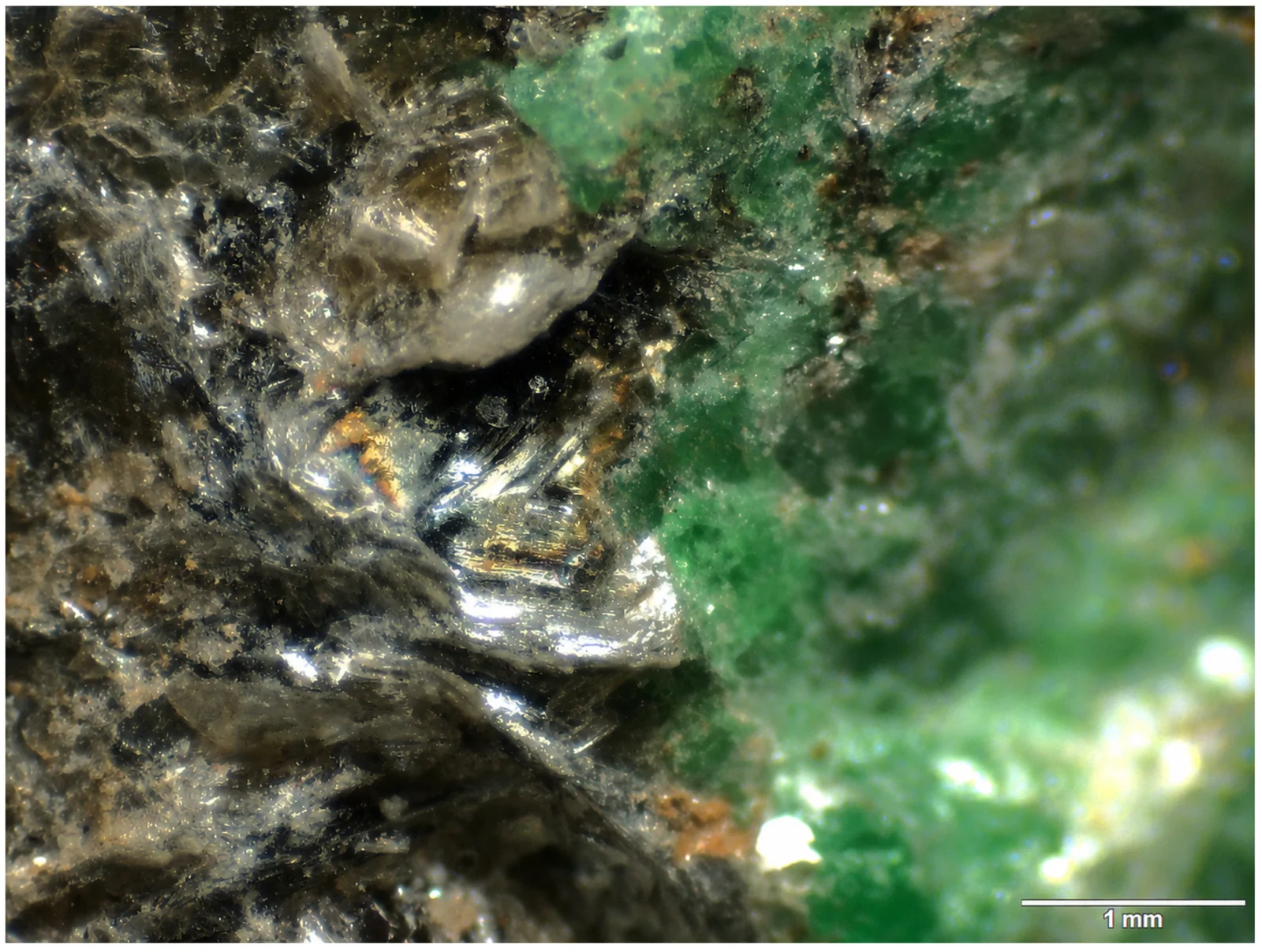
Microscopic appearance of the contact between green beryl and host rock. The boundary between green beryl and the surrounding mineral grains is irregular, with local fractures and host-rock fragments; scale bar = 1 mm.

**Figure 4:**
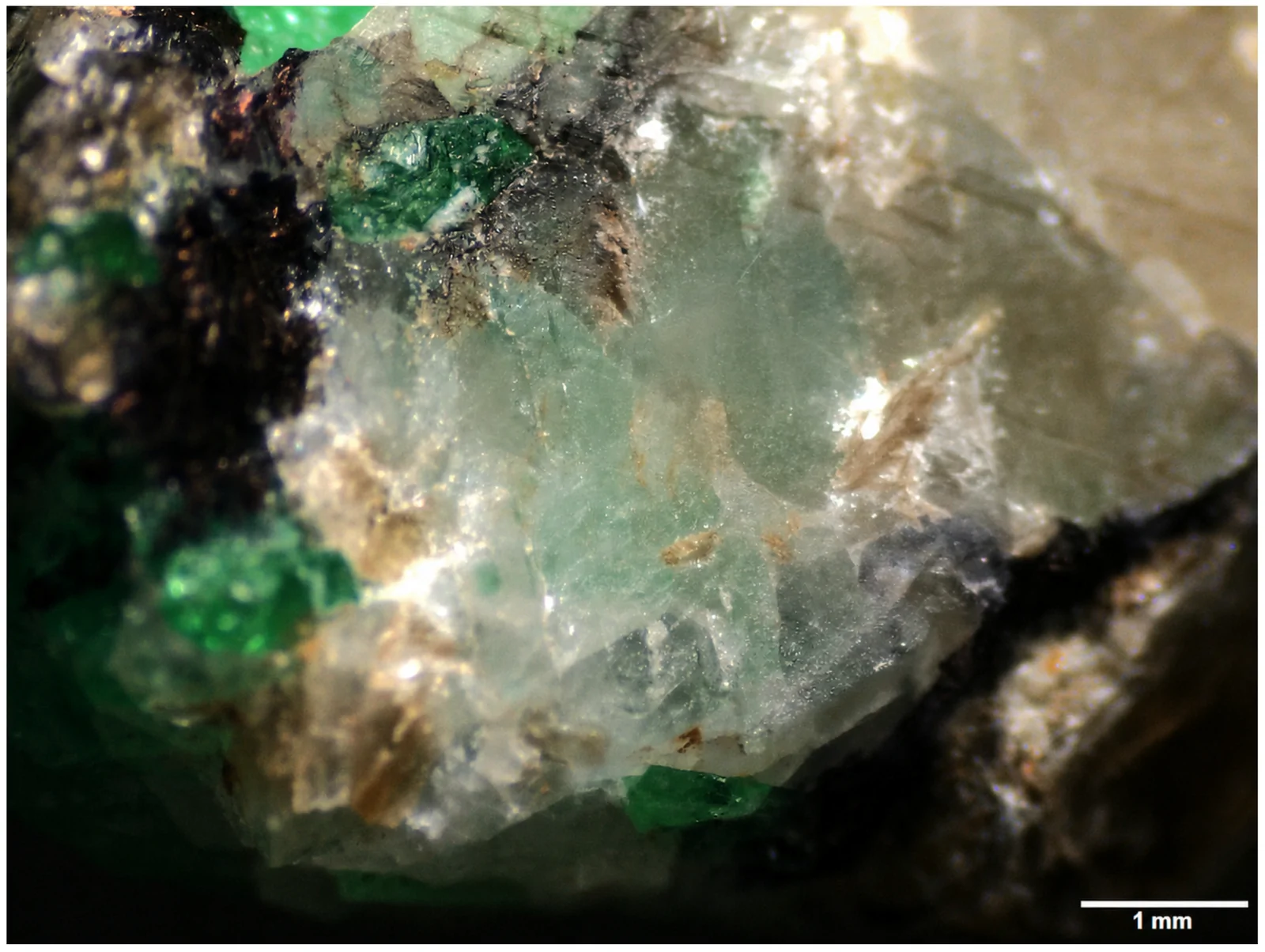
Microscopic appearance of a pale-green beryl aggregate in a host-rock-bearing specimen. Color and transparency are heterogeneous, and the aggregate has irregular contacts with the surrounding minerals; scale bar = 1 mm.

The samples were obtained from the North Muzart River area in Zhaosu County, Xinjiang, northwestern China. Ten natural green beryl specimens were selected, including three specimens attached to host rock. In samples M1–M3, the green beryl occurs mainly as irregular aggregates or locally developed hexagonal prismatic crystals. Crystal–host-rock boundaries are irregular, and fractures and fragments of host-rock minerals are locally visible (Figure 1). Detached crystals E1–E7 retain hexagonal prismatic outlines, natural crystal faces, and longitudinal striations parallel to the c-axis. They are generally of low transparency and range from pale to dark green (Figure 2). External morphology was examined with a GI-MP22 (Baoguang Technologies, Nanjing, China) gemological microscope equipped for photography. Figure 3 and Figure 4 show representative contact boundaries and local microscopic features of the host-rock-bearing samples. For consistency with the analytical scope of this study, the investigated specimens are designated as green beryl—specifically, natural V-rich green beryl rather than emerald; the term ‘emerald’ is retained only in general or literature-based discussion.

EDXRF analyses were performed at the Gemological Experimental Teaching Center, School of Gemology, China University of Geosciences (Beijing, China), using a Shimadzu EDX-7000 energy-dispersive X-ray fluorescence spectrometer (Shimadzu, Kyoto, Japan) for nondestructive semiquantitative analysis. Each sample was placed in a sample cup covered with Mylar film and analyzed under vacuum with a measurement diameter of 1 mm. Thirteen point analyses were obtained from the seven detached crystals, with one to three positions measured on each crystal depending on its size, surface flatness, and accessible analytical area. Element contents were calculated by the fundamental-parameter method and reported as semiquantitative oxide-equivalent contents. Because Be cannot be measured reliably by this method, the data were not used to reconstruct the complete stoichiometry of beryl but to compare the detectable compositional characteristics of V, Fe, and Cr. A matrix-specific quantitative detection limit for Cr_2_O_3_ was not established for the semiquantitative fundamental-parameter EDXRF analysis used in this study. Accordingly, ‘not detected’ is used qualitatively and should not be interpreted as indicating zero Cr concentration.

UV–Vis–NIR spectra were collected at the same center using a Shimadzu UV-3600 UV–Vis–NIR spectrophotometer (Shimadzu, Kyoto, Japan) in reflectance geometry over 200–900 nm with a data interval of 1 nm. The instrument software reported the spectra directly in absorbance units; these data are therefore treated here as apparent absorbance. The c-axis was identified from the natural crystal morphology, and spectra were collected from crystal surfaces approximately parallel or perpendicular to the c-axis. No polarizing accessory was used; these measurements therefore represent approximate orientation comparisons rather than polarized spectra.

Raman spectra were acquired at the same center using a HORIBA LabRAM HR Evolution confocal micro-Raman spectrometer (HORIBA Scientific, Palaiseau, France) with 532 nm excitation, a 50× long-working-distance objective, and a 600 grooves/mm grating. Spectra were collected over 100–4000 cm^−1^ with an integration time of 5 s and two accumulations at each analytical point. Data were recorded and processed using LabSpec 6 software. For the beryl-host characterization presented in this manuscript, two Raman spots were evaluated: one on E1 and one on E6. Both spots were selected from visually homogeneous green regions and deliberately positioned away from visible inclusions, fractures, and host-rock contacts to minimize interference from secondary phases.

FTIR spectra were collected from four selected detached crystals (E1, E3, E4, and E7) at the same center using a Bruker TENSOR 27 Fourier-transform infrared spectrometer (Bruker, Ettlingen, Germany) in transmission and reflectance modes. The spectral range was 8000–400 cm^−1^, the resolution was 4 cm^−1^, and each spectrum was accumulated over 32 scans using a 6 mm aperture.

## 3. Results

### 3.1. EDXRF Analysis

Thirteen EDXRF point analyses were obtained from the seven detached crystals (E1–E7) (Table 1). The V_2_O_5_-equivalent contents range from 0.562 to 4.616 wt.%, and the Fe_2_O_3_-equivalent contents range from 0.172 to 0.494 wt.%. Cr_2_O_3_ was not detected at any analytical point under the present EDXRF conditions. Variations in V_2_O_5_-equivalent content among crystals and between positions on the same crystal indicate a degree of compositional heterogeneity.

**Figure 5:**
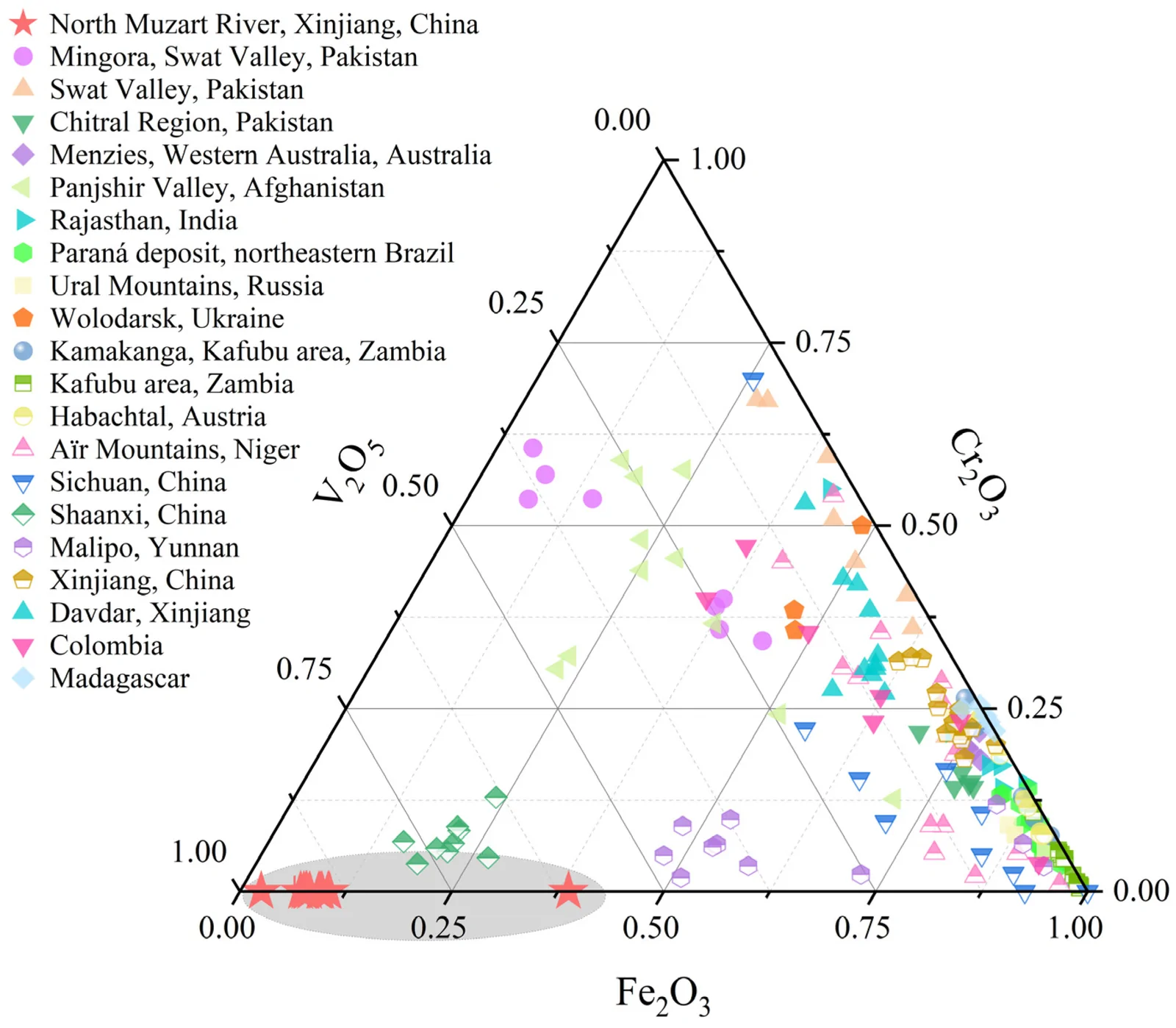
Normalized Fe_2_O_3_–V_2_O_5_–Cr_2_O_3_ ternary diagram for natural V-rich green beryl from the North Muzart River area and green beryl from other localities. Data for the North Muzart River samples are from the present EDXRF analyses; comparative data are from references [19,21,30,31,32,33,34,35,36,37,38,39,40,41,42,43,44,45,46,47,48,49]. The three oxide-equivalent components were normalized to Fe_2_O_3_ + V_2_O_5_ + Cr_2_O_3_ = 100%. Points for which Cr_2_O_3_ was not detected are plotted on the Cr_2_O_3_ = 0 boundary for visualization; their positions do not indicate that the actual Cr content is zero. The gray shaded ellipse is used only to highlight the distribution of the North Muzart River samples and has no statistical meaning.

To visualize the relative proportions of the three transition-metal components, the Fe_2_O_3_-, V_2_O_5_-, and Cr_2_O_3_-equivalent contents from the 13 EDXRF analyses were normalized and plotted together with literature data for green beryl from other localities in an Fe_2_O_3_–V_2_O_5_–Cr_2_O_3_ ternary diagram (Figure 5).

The North Muzart River data cluster near the V_2_O_5_ endmember, with a comparatively small Fe_2_O_3_ proportion. Because Cr_2_O_3_ was not detected under the present EDXRF conditions, the points are plotted on the Cr_2_O_3_ = 0 boundary as a visualization convention; this position does not imply that the actual Cr concentration is zero. Relative to the comparison dataset, the samples occupy a V-dominant field and are clearly separated from many Cr-dominant or Fe–Cr-rich samples.

**Figure 6:**
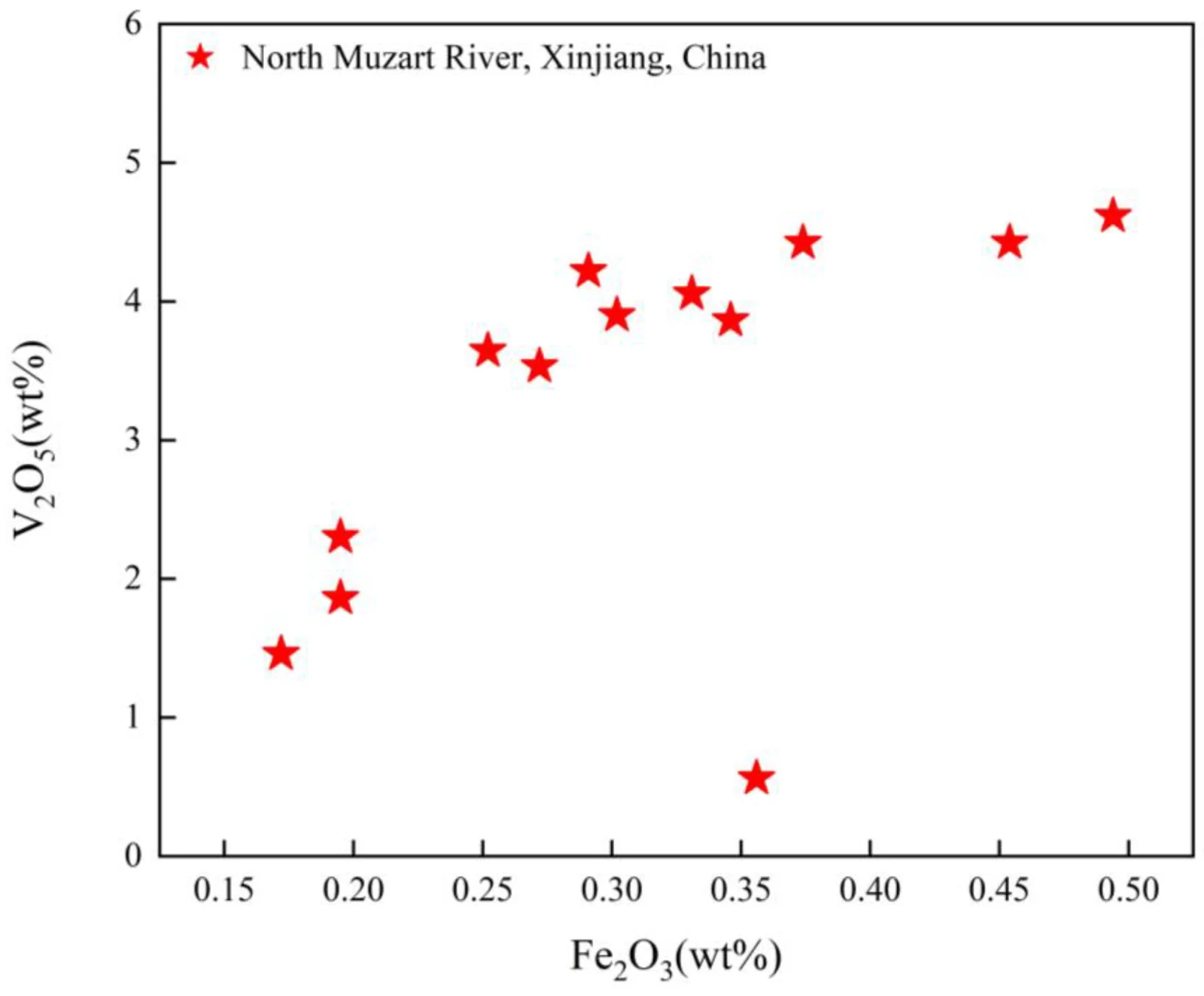
Binary plot of Fe_2_O_3_- and V_2_O_5_-equivalent contents in natural V-rich green beryl from the North Muzart River area. The dataset comprises 13 EDXRF point analyses, and both oxide contents are semiquantitative EDXRF equivalents.

The Fe_2_O_3_–V_2_O_5_ binary plot displays a broadly positive distribution, although E7-1 is a clear outlier and the remaining points retain appreciable scatter (Figure 6). Given the semiquantitative oxide-equivalent data and limited sample size, the plot is used only to show the compositional distribution and not to infer lattice-scale coupling between V and Fe.

### 3.2. UV–Vis–NIR Spectroscopy

**Figure 7:**
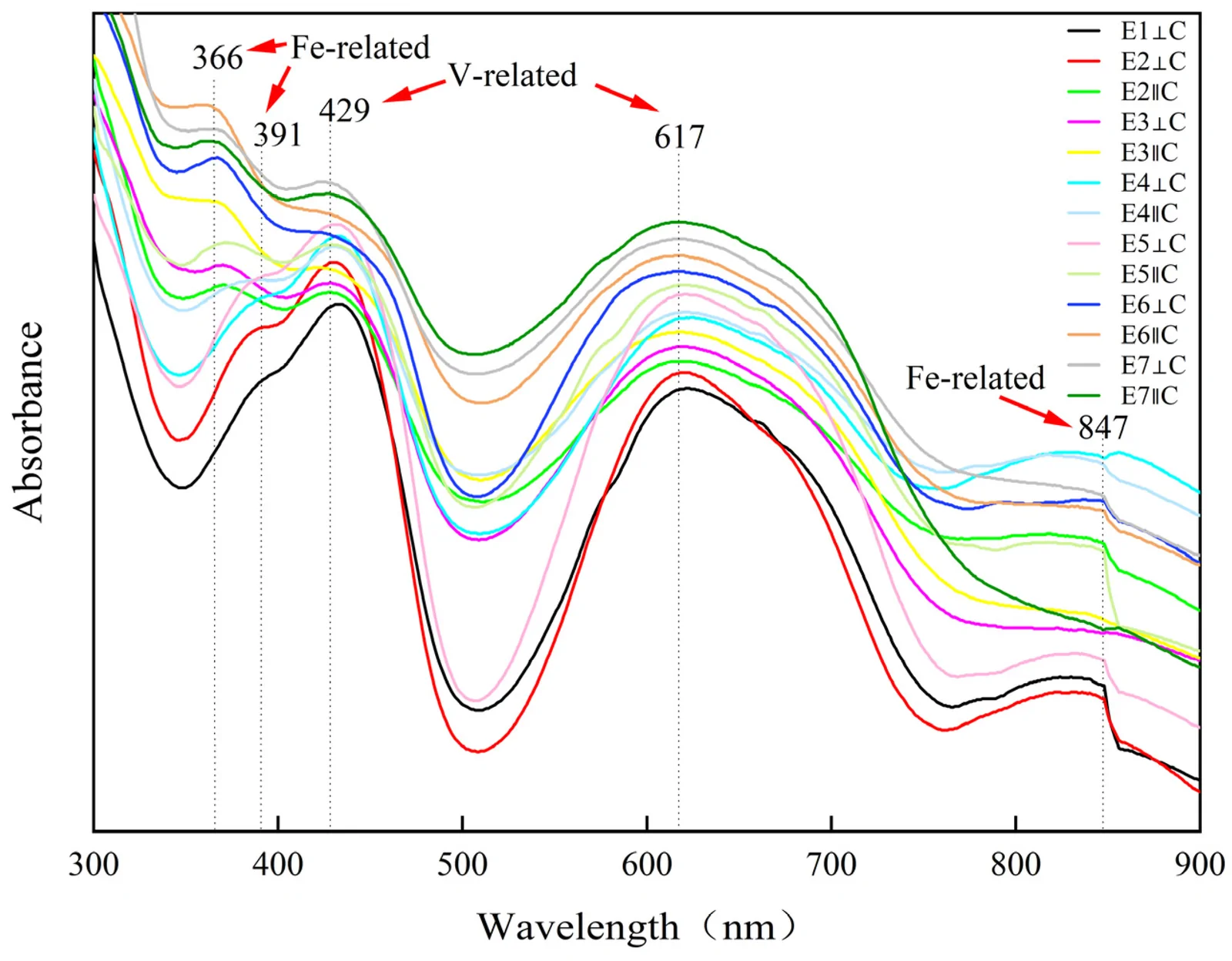
UV–Vis–NIR apparent absorbance spectra of natural V-rich green beryl from the North Muzart River area. The E1 spectrum was collected from a crystal surface approximately perpendicular to the c-axis; paired spectra for E2–E7 were collected from surfaces approximately parallel and perpendicular to the c-axis. Spectral annotations indicate tentative literature-based assignments rather than direct valence-state determinations in the present samples.

**Figure 8:**
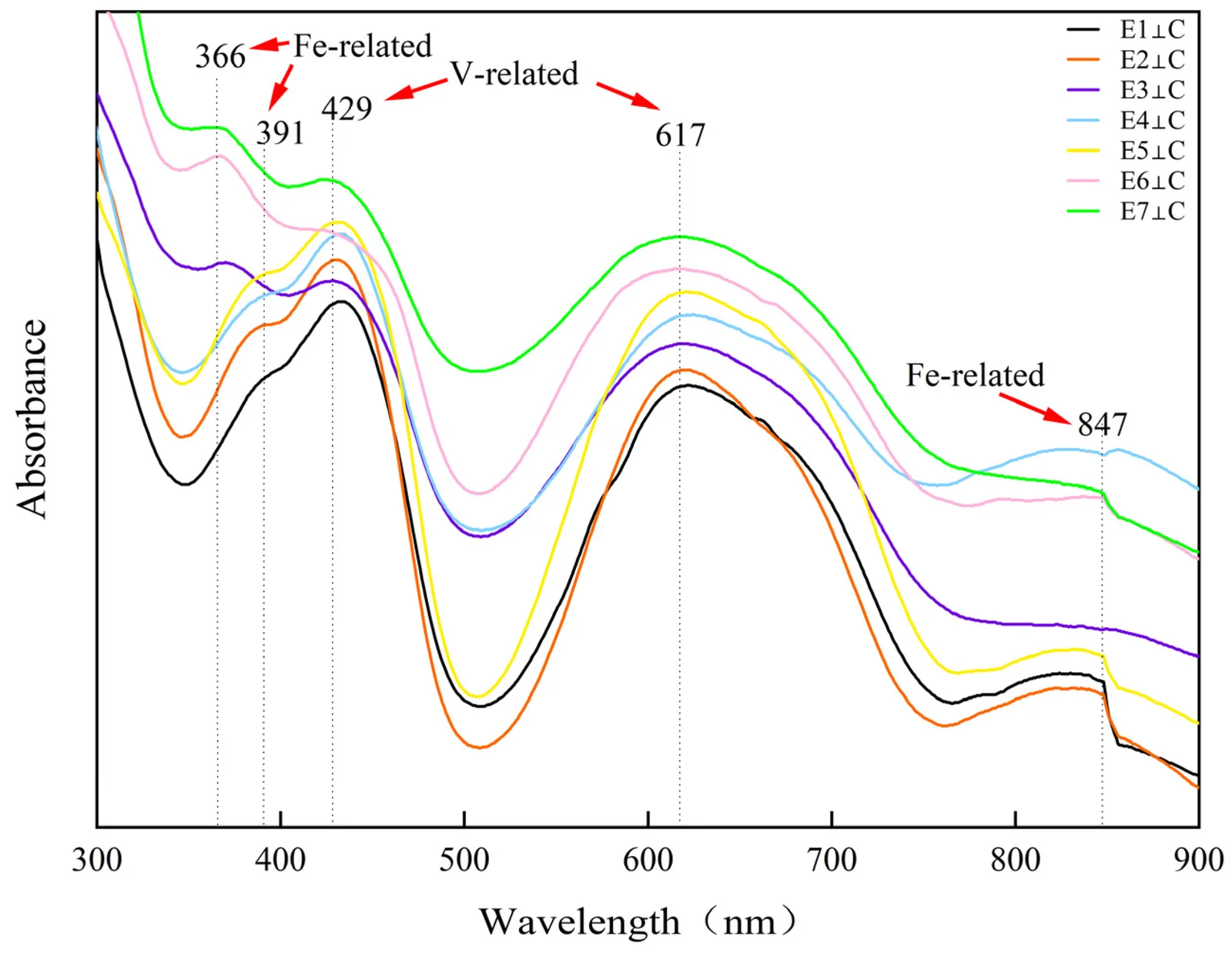
UV–Vis–NIR apparent absorbance spectra of natural V-rich green beryl from the North Muzart River area collected from crystal surfaces approximately perpendicular to the c-axis. Spectral annotations indicate tentative literature-based assignments rather than direct valence-state determinations in the present samples.

Thirteen UV–Vis–NIR spectra were obtained from the seven detached crystals. The E1 spectrum was collected from a surface approximately perpendicular to the c-axis, whereas paired spectra for E2–E7 were collected from surfaces approximately parallel and perpendicular to the c-axis. All spectra have similar broad-band profiles, with two principal bands recurring near 429 and 617 nm. Weaker responses occur near 366 and 391 nm, together with a weak, broad near-infrared response near 847 nm (Figure 7). The paired spectra have broadly similar principal band positions but differ in relative intensity, band shape, and baseline. These orientation designations are approximate and should not be interpreted as polarized measurements or as quantitative characterization of dichroism. To compare band positions under the same approximate orientation, the seven spectra collected from surfaces approximately perpendicular to the c-axis were plotted separately (Figure 8). The principal bands near 429 and 617 nm and the weaker responses near 366, 391, and 847 nm remain identifiable, demonstrating that the main band positions are reproducible under the same nominal orientation. Differences in relative intensity and baseline among the spectra may reflect variations in crystal thickness; surface flatness; transparency; fracture density; and, where present, minor surface residues. Accordingly, baseline differences are not interpreted quantitatively.

### 3.3. Raman Spectroscopy

**Figure 9:**
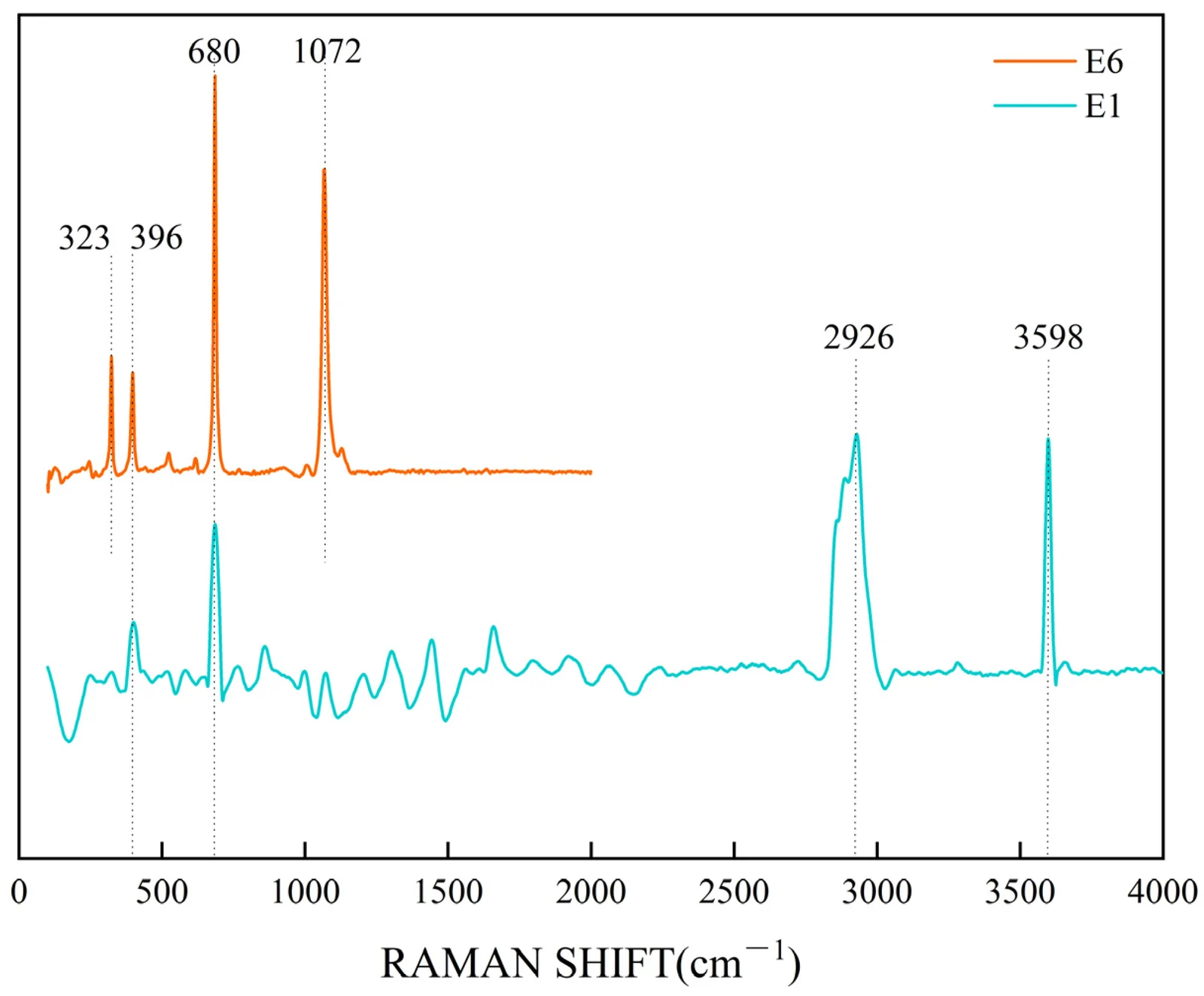
Raman spectra of natural V-rich green beryl samples E1 and E6 from the North Muzart River area.

The Raman spectrum of E6 contains characteristic bands near 323, 396, 680, and 1072 cm^−1^ (Figure 9). The bands near 323 and 396 cm^−1^ are associated with low-frequency lattice vibrations involving Al–O motion. The intense bands near 680 and 1072 cm^−1^ are characteristic of beryl and involve Be–O/silicate-ring-related vibrations and Si–O stretching, respectively [13,14]. This band set is consistent with the beryl framework at the analyzed E6 location. The high-wavenumber Raman spectrum of E1 contains a water-related band near 3598 cm^−1^, consistent with the type-II channel-water response observed by FTIR [13,15,16,28,50]. The E1 spectrum also contains a C–H stretching signal near 2926 cm^−1^, probably derived from surface organic residue or local contamination; it is not treated as an intrinsic structural feature of beryl. At the two selected analytical locations, the Raman spectra are consistent with the beryl framework, with no strong bands attributable to a secondary phase. This observation is restricted to the analyzed regions and does not imply phase purity of the entire specimens.

### 3.4. FTIR Spectroscopy

**Figure 10:**
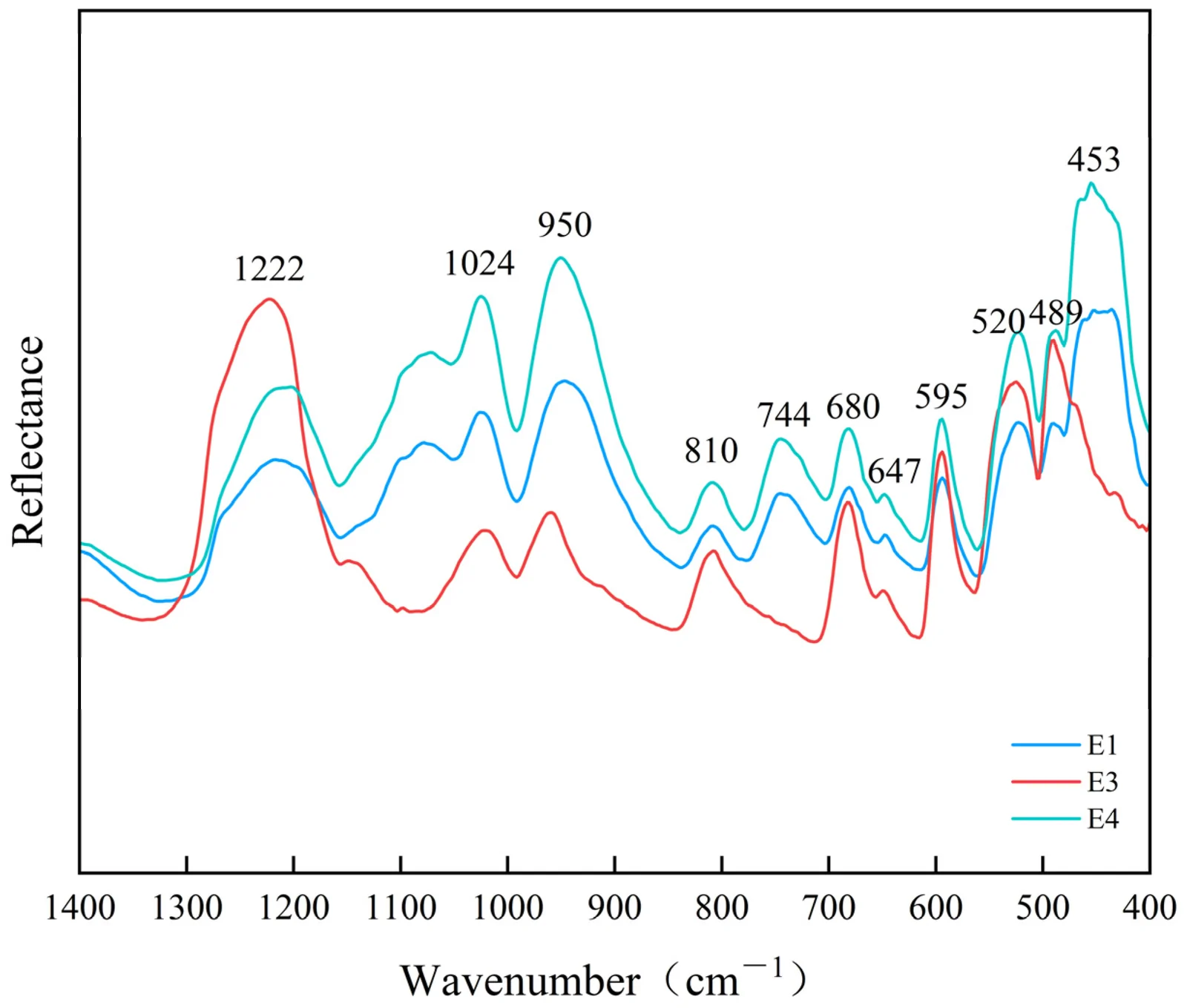
FTIR spectra of samples E1, E3, and E4 over 400–1400 cm^−1^.

**Figure 11:**
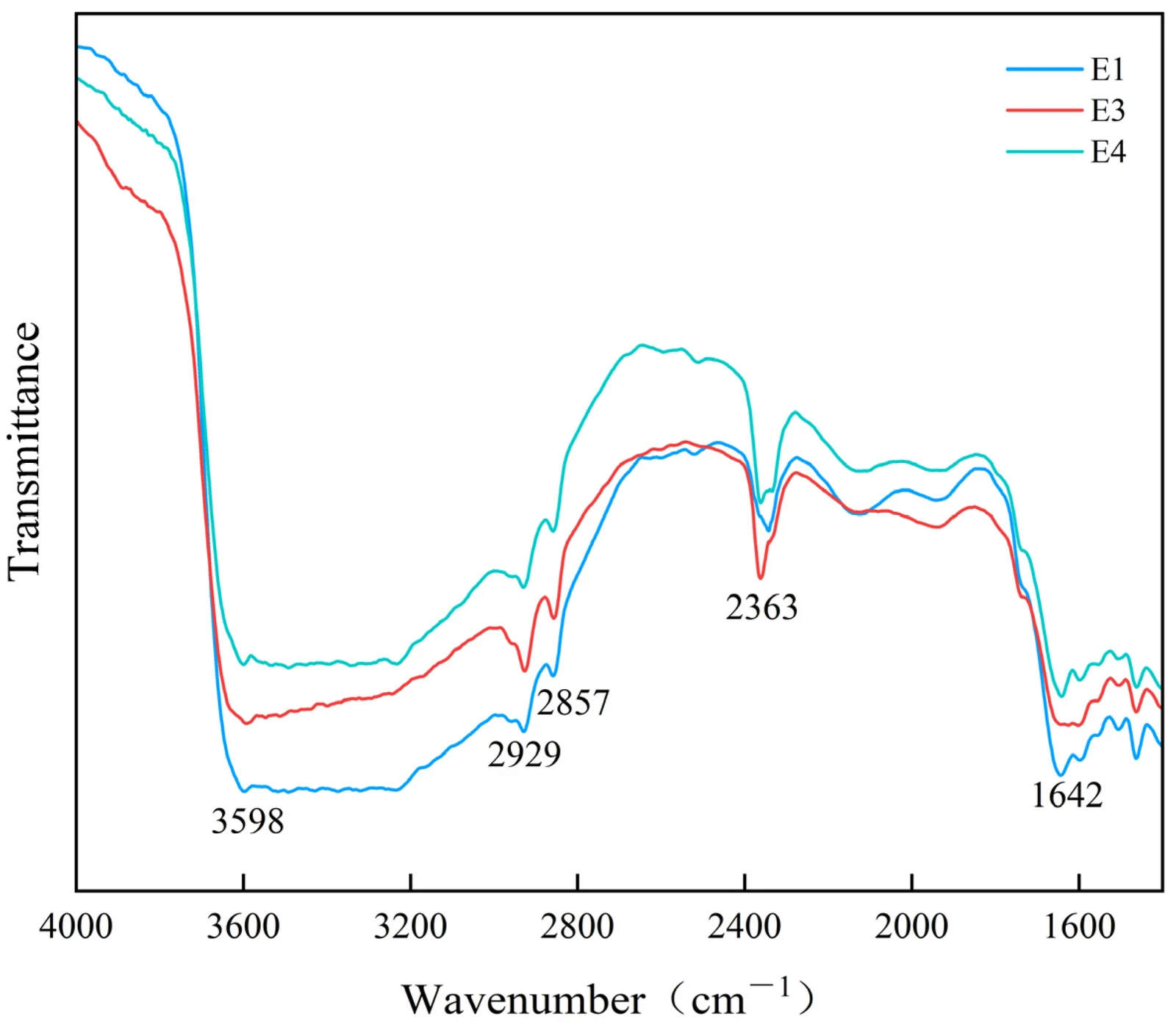
FTIR spectra of samples E1, E3, and E4 over 1400–4000 cm^−1^.

The FTIR spectra of E1, E3, and E4 display similar sets of beryl framework bands between 453 and 1222 cm^−1^, including vibrations related to Si–O, Si–O–Si, O–Si–O, and Be–O units, indicating that the samples retain the characteristic beryl framework (Figure 10). Water-related bands occur near 1642 and 3598 cm^−1^ (Figure 11). Features near 2857 and 2929 cm^−1^ may reflect C–H stretching or surface organic residues. The weak response near 2363 cm^−1^ is likely associated with environmental CO_2_ or CO_2_ in the optical path and is therefore not assigned to a structural component.

**Figure 12:**
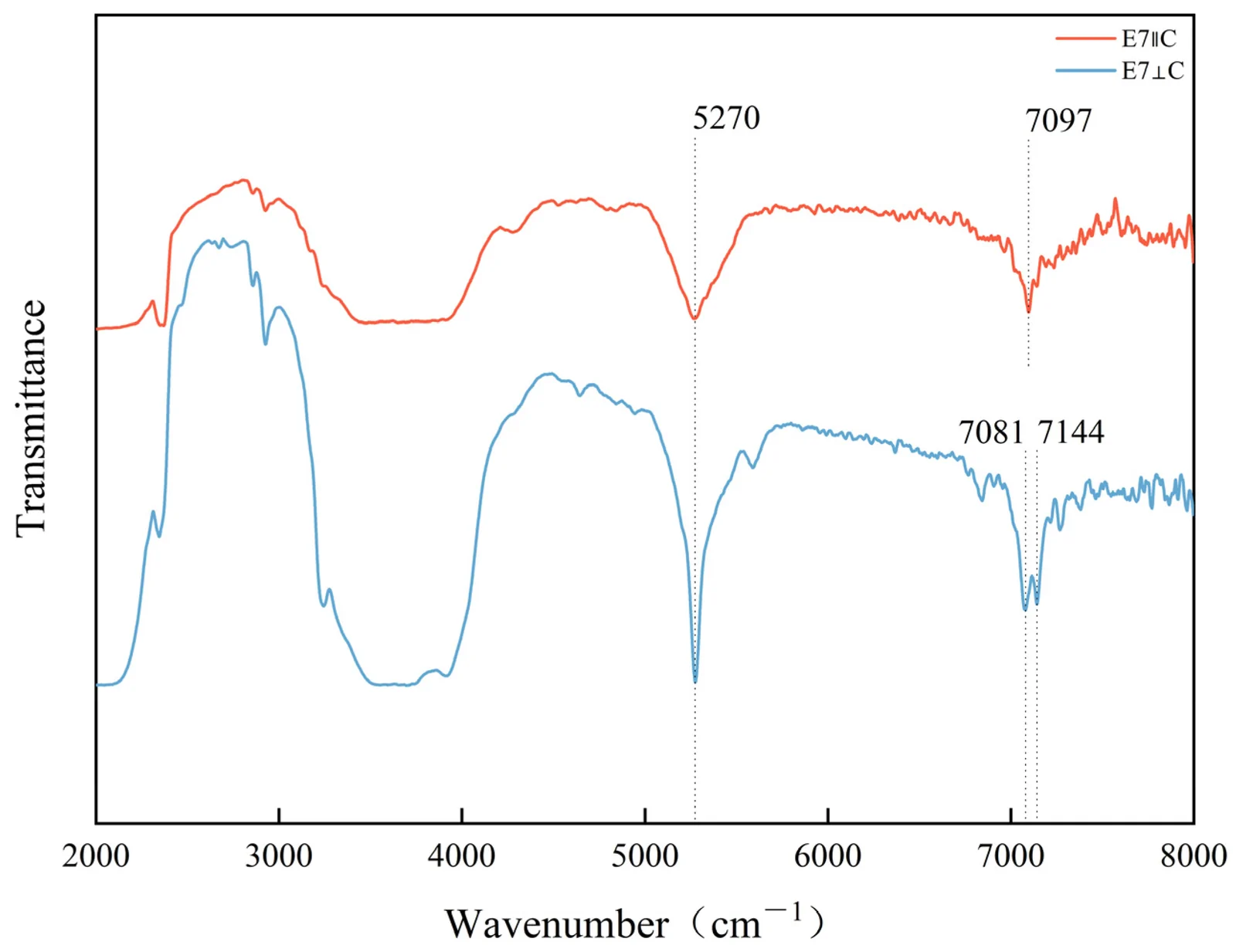
FTIR spectra of sample E7 over 2000–8000 cm^−1^, collected from crystal surfaces approximately parallel and perpendicular to the c-axis.

Sample E7 contains water-related combination or overtone absorptions near 5270, 7081–7097, and 7144 cm^−1^ (Figure 12). Based on previous studies of water in beryl channels, the bands near 1642, 3598, and 7081–7097 cm^−1^ are mainly consistent with type-II water, whereas the band near 5270 cm^−1^ may contain contributions from both type-I and type-II water; the weaker feature near 7144 cm^−1^ is associated with type-I water. Taken together, the channel-water features observed in the four FTIR-analyzed crystals are predominantly consistent with type-II water, with a weaker type-I contribution [5,15,16,17,28,50,51,52]. The principal bands and their preliminary assignments are summarized in Table 2.

## 4. Discussion

### 4.1. V-Dominant Composition and Undetected Cr: Constraints on V-Related Electronic Absorption

The principal visible absorption ranges of V^3+^ and Cr^3+^ overlap strongly in natural emerald, making their relative contributions difficult to determine from band positions alone [8,18,19,20,21]. EDXRF analysis of the North Muzart River samples indicates a distinctly V-dominant composition, with Cr not detected under the present analytical conditions; the Fe_2_O_3_–V_2_O_5_–Cr_2_O_3_ ternary plot likewise places the data close to the V_2_O_5_ endmember. The Fe_2_O_3_–V_2_O_5_ distribution is appreciably scattered and does not support a lattice-coupling relationship between the two components. Within this compositional context, the recurring broad bands near 429 and 617 nm are consistent with the characteristic visible absorptions commonly attributed to V^3+^ in beryl and are interpreted here as predominantly V-related [8,18,19,20,21]. This interpretation remains indirect and does not exclude a minor contribution from Cr below the EDXRF detection capability.

The weaker responses near 366, 391, and 847 nm may involve Fe-related centers and appear as additional absorption in the near-ultraviolet to short-wavelength visible region and in the near-infrared region [9,10,11,12,22,23]. When only the seven spectra collected from surfaces approximately perpendicular to the c-axis are compared, the principal bands remain near 429 and 617 nm, indicating that their positions do not arise from combining spectra obtained in different orientations.

EDXRF provides semiquantitative compositional information and does not directly determine the valence state, lattice site, or local coordination of V; the oxidation-state assignment is therefore indirect. In a qualitative crystal-field framework, if V occurs as V^3+^ (3d^2^) at the approximately octahedral Al site of beryl, broad visible d–d absorptions are expected. The present bands at ~617 and ~429 nm correspond to ~16,200 and ~23,300 cm^−1^, respectively. V-rich Malipo emeralds show comparable maxima at 432/611 nm (o-ray) and 425/644 nm (e-ray) [18], while V-bearing Nigerian beryl shows broad bands near 427 and 610 nm [20]. These comparisons support a predominantly V-related interpretation without independently establishing the V valence state. Representative absorption features of V-bearing natural and synthetic beryl are compared in Table 3.

### 4.2. Vibrational-Spectroscopic Features of the Beryl Framework and Channel Water

Raman spectra from the two selected green regions provide local phase confirmation of the beryl host and therefore support the spectroscopic interpretation, although they do not establish phase purity beyond the analyzed locations. The principal absorption bands near 429 and 617 nm are therefore more likely to originate from transition-metal-related centers within the analyzed beryl host than from a secondary phase [13,14].

The Raman spectrum of E1 contains a water-related response near 3598 cm^−1^, consistent with channel water. Across the four FTIR-analyzed crystals, the observed water-related bands are predominantly consistent with type-II water, accompanied by weaker type-I features [5,15,16,17,28,50,51,52].

The relative intensities of type-I and type-II water bands are influenced by channel cations and growth conditions. Because type-II H_2_O is associated with water molecules whose orientation is affected by nearby channel cations, its predominance provides crystal-chemical information on the channel environment [5,15,16,17,50,51]. However, this feature alone does not uniquely constrain the mineralizing-fluid composition or physicochemical conditions and is not used here as an independent genetic or geographic-origin indicator.

### 4.3. Significance of the North Muzart River Samples as a Natural Comparative System

Compared with emeralds in which Cr and V commonly coexist, the North Muzart River samples show a V-dominant composition, with Cr not detected under the present EDXRF conditions, and therefore provide a natural comparative system for examining V-related electronic absorption [18,20,21,30,34,35,37,38]. Raman and FTIR spectroscopy further establish the associated beryl-framework and channel-water background.

The samples can also be compared with experimentally V-doped beryl and V-bearing synthetic emerald, particularly with respect to the principal absorption bands near 429 and 617 nm [25,27]. In gemological characterization, this combination of compositional and spectroscopic features may serve as one component of a multi-parameter comparison with other green beryls and emeralds. However, these features are not sufficient on their own for geographic-origin determination or natural-versus-synthetic identification. For synthetic materials, the North Muzart River samples provide a natural reference for V-related absorption rather than a quantitative model for dopant concentration or crystal-growth conditions.

The crystals were not directionally cut and differ in thickness, surface condition, transparency, and fracture density. No polarizing accessory was used, and the EDXRF and spectroscopic analytical areas were not strictly coincident. The present comparisons are therefore limited to band position, band shape, and relative intensity. Absolute absorption coefficients were not calculated, and no quantitative relationships were established between V content and absorption intensity or between crystallographic direction and dichroism. Rigorous crystal-field analysis requires controlled orientation and polarized spectra, whereas quantitative concentration–absorption relationships additionally require thickness-normalized spectra and more precise composition data. The present study did not address crystal-growth processes or spatial V-concentration gradients and therefore cannot constrain the detailed incorporation of V or the physicochemical conditions of crystal growth.

## 5. Conclusions

Natural V-rich green beryl from the North Muzart River area is characterized by a V-dominant EDXRF composition, with Cr not detected under the present analytical conditions, and recurrent absorption bands near 429 and 617 nm. Their positions agree with V-related absorptions reported for V-rich beryl and are therefore interpreted as predominantly V-related, while the V^3+^ assignment remains indirect because EDXRF does not determine the oxidation state, crystallographic site, or local coordination of V and cannot exclude a minor contribution from Cr below the EDXRF detection capability. Raman spectra from the two selected green regions are consistent with the beryl host, and FTIR spectra of the four analyzed crystals show predominantly type-II channel-water features.

The main contribution of this study is to provide an integrated compositional and spectroscopic reference for an unusual natural V-dominant beryl system, including electronic absorption, framework vibrations, and channel-water characteristics. Its value is primarily comparative rather than quantitative because of the semiquantitative EDXRF data, variable crystal thickness, and only approximate crystallographic orientation. Future work combining valence-sensitive measurements, controlled polarized absorption spectroscopy, and higher-resolution compositional analysis could test V-site/valence assignments and concentration–absorption relationships more directly.

## Figures and Tables

**Table 1:** Semiquantitative EDXRF oxide-equivalent contents of natural V-rich green beryl from the North Muzart River area.

Cr_2_O_3_ (wt.%)	CuO (wt.%)	La_2_O_3_ (wt.%)	K_2_O (wt.%)	CaO (wt.%)	Cs_2_O (wt.%)	Fe_2_O_3_ (wt.%)	V_2_O_5_ (wt.%)	Al_2_O_3_ (wt.%)	SiO_2_ (wt.%)	Sample
n.d.	n.d.	0.013	0.089	0.171	0.185	0.195	2.301	16.446	77.984	E1-1
n.d.	0.007	0.027	0.084	0.067	0.111	0.374	4.423	15.865	78.794	E1-2
n.d.	0.007	0.048	n.d.	0.124	0.116	0.454	4.424	15.529	79.093	E1-3
n.d.	0.011	0.044	n.d.	0.057	0.086	0.272	3.532	17.187	78.695	E2-1
n.d.	0.014	0.048	n.d.	0.096	0.087	0.252	3.644	17.122	78.656	E2-2
n.d.	0.005	0.023	n.d.	0.101	0.092	0.302	3.901	16.439	79.019	E3
n.d.	n.d.	n.d.	0.142	0.454	0.453	0.172	1.459	15.229	81.896	E4-1
n.d.	n.d.	n.d.	0.109	0.219	0.557	0.195	1.861	15.196	81.704	E4-2
n.d.	n.d.	0.011	0.153	0.072	0.138	0.331	4.056	16.600	78.157	E5-1
n.d.	n.d.	0.008	0.113	0.050	0.121	0.291	4.218	15.866	78.726	E5-2
n.d.	n.d.	0.129	0.063	0.947	0.066	0.346	3.863	16.642	77.861	E6
n.d.	0.037	0.083	0.126	0.485	0.178	0.356	0.562	16.428	81.557	E7-1
n.d.	n.d.	0.010	n.d.	0.168	0.171	0.494	4.616	16.692	77.433	E7-2

n.d., not detected under the analytical conditions used in this study; this designation is qualitative and does not indicate zero concentration. A matrix-specific quantitative detection limit for Cr_2_O_3_ was not established for the present semiquantitative EDXRF analysis. Because Be cannot be measured reliably by this method and natural crystal surface conditions may affect semiquantitative results, the values in Table 1 are intended for comparison of detectable components rather than complete compositional or mineral-formula calculation.

**Table 2:** Principal FTIR bands of natural V-rich green beryl from the North Muzart River area and their preliminary assignments.

Preliminary Assignment	Band Position (cm^−1^)
Beryl framework vibrations (Si–O-, Si–O–Si-, O–Si–O-, and Be–O-related vibrations)	453–1222
Channel-water bending; mainly consistent with type-II water	~1642
Channel-water O–H stretching; mainly consistent with type-II water	~3598
Water-related combination band; may contain contributions from both type-I and type-II water	~5270
Water-related overtone/combination band; mainly consistent with type-II water	7081–7097
Weak type-I water-related overtone/combination band	~7144

**Table 3:** Comparison of representative absorption features of V-bearing natural and synthetic beryl.

Material/Study	Compositional Context	Representative Absorption Features (nm)	Interpretation/Comparison
North Muzart River (present study)	V-dominant; Cr_2_O_3_ not detected under present EDXRF conditions	~429, ~617; weaker ~366, ~391, ~847	Principal bands are interpreted as predominantly V-related; weaker features may involve Fe.
Malipo, China [18]	V-rich; Cr markedly lower than V	432/611 (o-ray); 425/644 (e-ray); ~395 shoulder; ~830	Closely comparable natural V-rich emerald; polarized spectra.
Nigeria [20]	V > Cr, although Fe is the predominant transition-metal component	~427, ~610; ~371, ~810; Cr-related features near 645–684 in some samples	Illustrates overlapping contributions from V-, Cr-, and Fe-related absorption in natural beryl
Biron V-bearing synthetic beryl [27]	V-bearing; Cr- and Fe-free	395, 430, 605, 645; ~680 sh.	Closer V-specific synthetic comparison; 395/430/605/645 nm attributed to V^3+^ at octahedral Al sites; ~680 nm shoulder uncertain.
Tairus hydrothermal synthetic emerald [27]	V- and Cu-bearing; Cr present only at relatively low levels	395, 430, 605, 645 (V-related); 750, 920, 1180 (Cu-related)	Provides a synthetic V-bearing comparison; the visible V-related bands are accompanied by Cu-related absorption.

Note: “Not detected” refers to the present EDXRF analytical conditions and does not indicate zero Cr concentration. Similar band positions do not necessarily indicate identical absorption centers. The Biron material is a V-bearing, Cr- and Fe-free synthetic research sample; the Tairus material is V- and Cu-bearing and is retained as an additional mixed-chromophore synthetic comparison.

## Data Availability

The original contributions presented in this study are included in the article. Further inquiries can be directed to the corresponding author.
